# Safety but limited efficacy of donor lymphocyte infusion for post-transplantation cyclophosphamide-treated patients

**DOI:** 10.1038/s41409-024-02312-4

**Published:** 2024-08-12

**Authors:** Krithika Shanmugasundaram, Scott Napier, Dimana Dimitrova, Anita Stokes, Jennifer Wilder, Amy Chai, Andrea Lisco, Megan V. Anderson, Irini Sereti, Gulbu Uzel, Alexandra F. Freeman, Christi McKeown, Jennifer Sponaugle, Ruby Sabina, Kamil Rechache, Mustafa A. Hyder, Jennifer A. Kanakry, Christopher G. Kanakry

**Affiliations:** 1 Center for Immuno-Oncology, Center for Cancer Research, National Cancer Institute, National Institutes of Health, Bethesda, MD USA; 2 Clinical Center, National Institutes of Health, Bethesda, MD USA; 3 HIV Pathogenesis Section, Laboratory of Immunoregulation, National Institute for Allergy and Infectious Disease, National Institutes of Health, Bethesda, MD USA; 4 Laboratory of Clinical Immunology and Allergy and Infectious Diseases, National Institutes of Health, Bethesda, MD USA

**Keywords:** Cancer immunotherapy, Haematological cancer

## Abstract

The therapeutic efficacy of donor lymphocyte infusions (DLIs) given after allogeneic hematopoietic cell transplantation (HCT) is limited by risk of graft-versus-host disease (GVHD). Post-transplantation cyclophosphamide (PTCy) effectively prevents severe GVHD, but there are limited data on outcomes of DLIs given to PTCy-treated patients. We reviewed 162 consecutive PTCy-treated patients transplanted between 2015–2022 within the Center for Immuno-Oncology at the National Cancer Institute. Of 38 DLIs given to 21 patients after 22 HCTs, few DLIs were associated with toxicities of acute GVHD (7.8%), cytokine release syndrome (CRS, 7.8%), or chronic GVHD (2.6%), and all occurred in those receiving serotherapy-containing pre-HCT conditioning (50% of HCTs). Seven DLIs resulted in complete response (18.4%), with 5 of these given after HCTs using serotherapy-containing conditioning. Excluding infectious indications, complete response to DLIs given after transplants with versus without serotherapy-containing pre-HCT conditioning were 30% and 4.3%, respectively. Two patients received DLI for infection and experienced complete resolution without GVHD or CRS, although the efficacy cannot be definitively attributable to the DLI. DLIs given to PTCy-treated patients had low toxicity but limited efficacy, although pre-HCT serotherapy may modulate both toxicity and response. Novel strategies are needed to enhance the therapeutic efficacy of post-transplant cellular therapies without aggravating GVHD.

## Introduction

Malignancy relapse remains the leading cause for failure of allogeneic hematopoietic cell transplantation (HCT) [1]. Donor lymphocyte infusions (DLIs) remain a major strategy to treat and prevent post-transplant relapse, with the highest response rate seen in chronic-phase chronic myelogenous leukemia (CML) (60–78%) [2–6]. Long-term responses also are seen in a minority of patients with blast-phase CML (17%) or acute myeloid leukemia (AML) (14–37%), but are very low in acute lymphoblastic leukemia (ALL) (<16%) or non-Hodgkin lymphoma (<10%) [3, 4, 7, 8, 9]. DLIs also are commonly used to treat mixed chimerism and sometimes are used to treat post-transplant infections [6, 10]. Nevertheless, even with limited response, risk for graft-versus-host disease (GVHD) after DLI is quite high with incidences ranging from 41–60% [3, 4, 7, 8, 9]. Chance of response is improved when disease-targeted, lymphodepleting therapy is given prior to DLI, although toxicity of treatment including risk of GVHD is exacerbated [9, 11–14].

Post-transplantation cyclophosphamide (PTCy) has had a marked impact on the field of allogeneic HCT by reducing the risks of severe acute and chronic GVHD, in part by facilitating rapid recovery of regulatory T cells (T_regs_) [15–19]. Moreover, preclinical data from our group have shown that PTCy rapidly induces suppressive mechanisms within 24 h that are sufficient to prevent GVHD induction by subsequent donor T-cell infusions [15, 17], with this effect dependent on Foxp3^+^ T_regs._ [20] Indeed, other groups have found that the risk for GVHD from DLI in PTCy-treated patients appears more modest than that seen with conventional calcineurin-inhibitor-based GVHD prophylaxis [3, 9, 12, 13, 21–24] even when using HLA-partially-mismatched donors.

These preclinical and clinical data have prompted us to be less hesitant in administration of DLI to PTCy-treated patients when the clinical need arises. Here, we reviewed outcomes of all PTCy-treated patients receiving therapeutic DLI on all HCT protocols in the Center for Immuno-Oncology at the National Cancer Institute from 2015 to 2022.

## Materials and methods

### Patients

All patients treated across all PTCy-based HCTs not containing prophylactic DLI within the Experimental Transplantation and Immunotherapy Branch/Center for Immuno-Oncology at the National Cancer Institute between December 1, 2015 and September 30, 2022 were included in this study. Decisions on dosing and preparative therapy for therapeutic DLI were at the discretion of the treating physician. The database was locked on March 21, 2023.

### Transplant regimens

Conditioning intensity was defined as per consensus criteria [25]. Reduced-intensity conditioning was with (1) pentostatin, low-dose hyper fractionated cyclophosphamide, and two days of busulfan [26] with or without two days of equine anti-thymocyte globulin (ATG) given on days −14 and −13, (2) fludarabine, cyclophosphamide, and 400 cGy total body irradiation, or (3) alemtuzumab (days −14 to −12), fludarabine, and melphalan. Non-myeloablative conditioning was achieved with (1) pentostatin and cyclophosphamide or (2) alemtuzumab (days −21 to −17), cyclophosphamide, and fludarabine. Myeloablative conditioning was with four days of busulfan and fludarabine [27].

Alemtuzumab has a half-life of 12–21 days [28, 29] and thus given between days −14 and −12 is expected to provide significant in vivo T-cell depletion of donor cells. By contrast, equine ATG has a half-life of 5.7 days [30] and was specifically given on days −14 and −13 in an attempt to decrease the extent of in vivo T-cell depletion of donor T cells [31] (i.e., provide primarily host in vivo T-cell depletion).

### Outcomes definitions

Indications for DLI were categorized into the following: delayed engraftment, mixed chimerism, poor immune reconstitution/infection, and relapse/progression of malignant disease.

Primary graft failure was defined as failure to achieve engraftment, which in turn was defined as achieving both neutrophil recovery (absolute neutrophil count ≥500 cells/µl for 3 consecutive days) and donor myeloid chimerism ≥5%. Secondary graft failure was defined as achieving initial engraftment but subsequently having donor myeloid chimerism decline to <5% on subsequent measurements. For those that received DLI for delayed engraftment, which was defined as lack of engraftment by day +28 after transplant, only complete response was evaluated, which was defined as achieving engraftment.

Chimerism by short tandem repeats (STR) was determined on CD3^+^ and CD33^+^ bead-selected peripheral blood mononuclear cells as per the NIH Clinical Center Department of Laboratory Medicine. Mixed chimerism was defined as myeloid chimerism ≥5% and <95% or donor CD3 chimerism <95%. Patients were reported as having incomplete response after DLI if chimerism remained stable (did not decrease ≥10%) or if it rose to <95%, while complete response was defined as chimerism increasing to ≥95% in both CD3 and myeloid compartments. No response was considered to have occurred if there was >10% decrease in either CD3 or myeloid chimerism or if second HCT was required.

For DLIs administered due to poor immune reconstitution/infection, complete response was considered to have been achieved if at any point after the DLI, there was complete resolution of the infection or virally-associated neoplasm following DLI. Incomplete response was determined if there was objective regression or stability of virally-associated neoplasm but incomplete response was not assessed for other types of infection.

For DLIs given for relapse or progression of the disease, complete response was considered to have been achieved if complete remission was achieved. Disease control without complete remission (i.e., partial response or stable disease) was considered an incomplete response. Response to DLI was considered inevaluable if, in the absence of response, subsequent cellular infusion or death occurred within 27 days.

One patient received 5 DLIs in the setting of uncontrolled human papillomavirus (HPV)-associated recurrent invasive and in situ squamous cell carcinoma (SCC) of the hands and feet as well as anogenital condylomas but he also had mixed chimerism, and thus he was evaluated separately for response for each of these two indications for all five of these DLIs.

CRS grading was by ASTCT consensus criteria [32], acute GVHD grading was by Keystone Criteria [33], and chronic GVHD diagnosis and grading was by NIH Consensus Criteria [34].

## Results

### Transplant characteristics

A total of 162 patients received 174 PTCy-based HCTs during this time period. Of these patients, 21 received at least one DLI after 22 PTCy-based transplants and were included in this analysis (Table 1). The median patient age was 27 years (range 4–67). Indication for HCT was either to cure malignancy (*n* = 9), primary immunodeficiency (*n* = 8), or both (*n* = 5). Fourteen transplants used an HLA-haploidentical donor, five an HLA-matched-unrelated donor, one an HLA-mismatched-unrelated donor, and two an HLA-matched-sibling donor. Eleven transplants (50%) evaluated in this study used serotherapy (alemtuzumab [*n* = 3] or equine ATG [*n* = 8]) as a component of the transplant conditioning regimen.

**Table 1:** Recipient, transplantation, and donor lymphocyte infusion (DLI) characteristics.

	Total HCTs with DLI (*n* = 22)	Haplo donor (*n* = 14)	MUD (*n* = 5)	mMUD (*n* = 1)	MSD (*n* = 2)
Male recipient, *n*	12	8	2	0	2
Recipient age, median (range), years	27 (4–67)	24.5 (4–67)	11 (6–37)	27	35.5 (27–44)
Donor age, median (range), years	31 (17–66)	40 (17–66)	30 (26–43)	29	31 (26–36)
Female donor/male recipient, *n*	3	2	0	0	1
Indications for transplant, *n*^a^					
Malignancy	14	10	1	1	2
AML	1	1	0	0	0
B-ALL	1	1	0	0	0
T-cell lymphoma/leukemia	8	5	1	1	1
B-cell NHL	3	3	0	0	0
Histiocytic sarcoma	1	0	0	0	1
Primary immunodeficiency	13	9	4	0	0
CMV serostatus, donor/recipient, *n*					
+/+	5	4	0	0	1
+/−	6	4	2	0	0
−/+	3	2	0	0	1
−/−	5	2	2	1	0
+/unknown^b^	2	1	1	0	0
−/unknown^b^	1	1	0	0	0
HCT conditioning regimen intensity, *n*					
Myeloablative	2	2	0	0	0
Reduced-intensity	16	10	4	0	2
Non-myeloablative	4	2	1	1	0
HCT graft source, *n*					
T-cell-replete bone marrow	11	7	3	0	1
T-cell-replete peripheral blood stem cells	11	7	2	1	1
HCT GVHD prophylaxis regimen, *n*					
PTCy 50 mg/kg/day on days +3 and +4, MMF, tacrolimus	3	1	2	0	0
PTCy 50 mg/kg/day on days +3 and +4, MMF, sirolimus	13	8	3	0	2
PTCy 50 mg/kg/day on days +3 and +4, sirolimus	1	0	0	1	0
PTCy 50 mg/kg/day on days +3 and +4, tacrolimus, ruxolitinib	1	1	0	0	0
PTCy 25 mg/kg/day on days +3 and +4, MMF, sirolimus	3	3	0	0	0
PTCy 25 mg/kg/day on day +4 only, MMF, sirolimus	1	1	0	0	0
Time to 1st DLI post-HCT in days, *n*					
31–60	7	6	1	0	0
61–90	6	5	0	1	0
91–120	0	0	0	0	0
>120	9	3	4	0	2
Total number of DLI doses received, *n*					
1	13	9	1	1	2
2	5	3	2	0	0
3	2	2	0	0	0
4	1	0	1	0	0
5	1	0	1	0	0

Characteristics (All DLIs)	Total DLIs (*n* = 38)	Haplo donor (*n* = 21)	MUD (*n* = 14)	mMUD (*n* = 1)	MSD (*n* = 2)
Indication for DLI, *n*^c^					
Mixed donor chimerism	25	13	12	0	0
Delayed primary engraftment^d^	2	2	0	0	0
Relapse or progression of hematologic malignancy	9	4	2	1	2
Infection/poor immune reconstitution	7	2	5	0	0
Conditioning pre-DLI, *n*					
None	28	15	11	1	1
Steroids only	5	3	2	0	0
Chemotherapy only	1	0	1	0	0
Chemotherapy + steroids	4	3	0	0	1
DLI cryopreserved or fresh, *n*					
Cryopreserved	29	14	13	1	1
Fresh	9	7	1	0	1
T-cell DLI doses (CD3+ cells/kg), *n*					
1 × 10^5^	3	3	0	0	0
5–6 × 10^5^	12	9	3	0	0
1 × 10^6^	10	3	6	1	0
2 × 10^6^	4	3	1	0	0
5 × 10^6^	3	2	1	0	0
1 × 10^7^	6	1	3	0	2
Toxicity of DLI, *n*					
Acute GVHD, any grade	3	2	1	0	0
Chronic GVHD	1	1	0	0	0
Cytokine release syndrome	3	3	0	0	0
Response to DLI, *n*					
Graft failure in spite of DLI(s) (*n* = 14 HCTs)	6	5	1	0	0
Attainment of full donor myeloid chimerism post-DLI (*n* = 19)^e^	1	1	0	0	0
Attainment of full donor T-cell chimerism post-DLI (*n* = 24)	3	3	0	0	0
Stability of chimerism (*n* = 27)	15	5	10	0	0
Complete remission of malignancy (*n* = 9)	1	0	0	1	0
Mixed response/partial malignancy control (*n* = 9)	3	2	0	0	1
Control of infection (*n* = 7)	7	2	5	0	0
No significant clinical effect	13	8	4	0	1

*HCT* hematopoietic cell transplantation, *Haplo* HLA-haploidentical, *MUD* 10/10 HLA-matched-unrelated donor, *mMUD* 7/8 HLA-matched-unrelated donor, *MSD* HLA-matched sibling donor, *AML* acute myeloid leukemia, *B-ALL* B-cell acute lymphoblastic leukemia, *NHL* non-Hodgkin leukemia, *CMV* cytomegalovirus, *GVHD* graft-versus-host disease, *PTCy* post-transplantation cyclophosphamide, *MMF* mycophenolate mofetil, *DLI* donor lymphocyte infusion.

^a^One patient with PTEN deficiency had both peripheral T-cell lymphoma and B-cell NHL, and 4 other patients were transplanted to treat malignancy in the setting of underlying primary immunodeficiency.

^b^Three patients who had been on chronic IVIG therapy prior to evaluation for transplant.

^c^One patient received 5 DLIs for both mixed chimerism and infection.

^d^Patients who had partial donor chimerism but failed to have ANC recovery ≥ 500

^e^One patient with full donor myeloid chimerism post-DLI had insufficient lymphocyte count for measurement of donor T-cell chimerism prior to death (transplant-related mortality).

### DLI characteristics

Thirty-eight DLIs were administered following these 22 HCTs (Table 2). The median time to DLI from HCT was 153 days (range 31–2411 days). Nine of the 22 HCTs were followed by more than one DLI. The primary indication for each DLI was as follows: mixed chimerism (*n* = 20), relapse or progression of hematologic malignancy (*n* = 9), poor immune reconstitution or uncontrolled infection (*n* = 7), and delayed primary engraftment (*n* = 2), although, as above, one patient received 5 DLIs for the secondary indication of mixed chimerism.

**Table 2:** Characteristics and Outcomes of Each Donor Lymphocyte Infusion.

Patient ID	Disease type	DLI #	Donor type	Dose	Cryo/Fresh	Pre-DLI lymphodepletion	Pre-HCT serotherapy	Achieved full chimerism		Time to response (days)	Acute GVHD	Chronic GVHD	CRS	Graft failure despite DLI
**MIXED CHIMERISM**								Myeloid	T-cell					
1	IEI (ADA2 deficiency)	1	MUD	5 × 10^6^/kg	cryo	Steroids	YES (ATG)	N	N	-	N	N	N	YES
2^a^	IEI (Idiopathic CD4+ lymphopenia)	1	MUD	5 × 10^5^/kg	cryo	N	N	N/A	N	-	N	N	N	N
		2	MUD	1 × 10^6^/kg	cryo	N	N	N/A	N	-	N	N	N	N
		3	MUD	1 × 10^6^/kg	cryo	N	N	N/A	N	-	N	N	N	N
		4	MUD	1 × 10^6^/kg	cryo	N	N	N	N	-	N	N	N	N
		5	MUD	1 × 10^6^/kg	cryo	N	N	N	NE	-	N	N	N	N
3	IEI (IRF8 mutation)	1	MUD	2 × 10^6^/kg	fresh	N	N	N	N	-	N	N	N	N
		2	MUD	1 × 10^7^/kg	cryo	N	N	N	N	-	N	N	N	N
4	IEI (IFNGR1 deficiency)	1	MUD	5 × 10^5^/kg	cryo	N	N	N	N	-	N	N	N	N
		2	MUD	5 × 10^5^/kg	cryo	N	N	N	N	-	N	N	N	N
		3	MUD	1 × 10^6^/kg	cryo	N	N	N	N	-	N	N	N	N
		4	MUD	1 × 10^6^/kg	cryo	N	N	N	N	-	N	N	N	N
5.1	IEI (Activated PI3K delta syndrome)/EBV+ DLBCL	1	haplo	1 × 10^5^/kg	fresh	N	N	N	N	-	N	N	N	N
		2	haplo	5 × 10^5^/kg	cryo	N	N	N	N	-	N	N	N	N
		3	haplo	5 × 10^5^/kg	cryo	N	N	N	N	-	N	N	N	YES
5.2	IEI (Activated PI3K delta syndrome)/EBV- DLBCL	1	haplo	5 × 10^5^/kg	cryo	N	YES (ALE)	N/A	YES	13	N	N	YES	N
6	IEI (PTEN deficiency)/Peripheral T-cell lymphoma and EBV+ lymphomatoid granulomatosis	1	haplo	5 × 10^5^/kg	fresh	N	N	N	N	-	N	N	N	N
		2	haplo	2 × 10^6^/kg	cryo	N	N	N	N	-	N	N	N	YES
7	IEI (Activated PI3K delta syndrome)	1	haplo	5 × 10^6^/kg	cryo	N	YES (ALE)	N/A	YES	14	YES	N	YES	N
8	IEI (NFKB1 haploinsufficiency)	1	haplo	1 × 10^6^/kg	fresh	N	N	N	N	-	N	N	N	N
		2	haplo	5 × 10^6^/kg	cryo	N	N	N	N	-	N	N	N	YES
9	B-cell acute lymphoblastic leukemia	1	haplo	1 × 10^5^/kg	fresh	Steroids	N	N/A	N	-	N	N	N	N
10	Acute myeloid leukemia	1	haplo	5 × 10^5^/kg	fresh	Steroids	N	N/A	N	-	N	N	N	N
		2	haplo	2 × 10^6^/kg	cryo	N	N	N	N	-	N	N	N	YES
11	IEI (unspecified)/Chronic active EBV, T-cell type	1	haplo	5 × 10^5^/kg	cryo	Steroids	YES (ATG)	N/A	YES	105	YES	N	N	N
**DELAYED PRIMARY ENGRAFTMENT**														
12	IEI (MAGT1 deficiency)	1	haplo	5 × 10^5^/kg	fresh	N	N	YES	NE	5	N	N	N	N
13	IEI (unspecified)/Anaplastic large cell lymphoma	1	haplo	6 × 10^5^/kg	cryo	N	YES (ATG)	NE	NE	-	N	N	YES	YES
**RELAPSE**														
	Malignancy							Best response	Response at last follow-up					
14	Anaplastic large cell lymphoma	1	MUD	1 × 10^7^/kg	cryo	Chemo	YES (ATG)	PD	PD	-	N	N	N	
		2	MUD	1 × 10^7^/kg	cryo	Steroids	YES (ATG)	PD	PD	-	YES	N	N	
15	Histiocytic sarcoma	1	MSD	1 × 10^7^/kg	fresh	N	N	IR	PD	-	N	N	N	
16	NK/T-cell lymphoma	1	MSD	1 × 10^7^/kg	cryo	Chemo/Steroids	YES (ATG)	PD	PD	-	N	N	N	
17	Adult T-cell lymphoma/leukemia	1	haplo	1 × 10^6^/kg	cryo	Chemo/Steroids	YES (ATG)	PD	PD	-	N	N	N	
18	Hepatosplenic γδ T-cell lymphoma	1	haplo	1 × 10^6^/kg	cryo	N	YES (ATG)	IR	IR	-	N	N	N	
		2	haplo	2 × 10^6^/kg	cryo	Chemo/Steroids	YES (ATG)	IR	IR	-	N	N	N	
		3	haplo	1 × 10^7^/kg	cryo	Chemo/Steroids	YES (ATG)	PD	PD	-	N	N	N	
19	Cutaneous T-cell lymphoma	1	mMUD	1 × 10^6^/kg	cryo	N	YES (ALE)	CR	CR	37	N	N	N	
**INFECTION**														
20	Cytomegalovirus pneumonitis	1	haplo	1 × 10^5^/kg	fresh	N	N	CR	CR	42	N	N	N	
21	Adenovirus viremia	1	haplo	5 × 10^5^/kg	cryo	N	YES (ATG)	CR	CR	20	N	YES	N	
2^a^	HPV-associated squamous cell carcinoma	1	MUD	5 × 10^5^/kg	cryo	N	N	IR	IR	-	N	N	N	
		2	MUD	1 × 10^6^/kg	cryo	N	N	IR	IR	-	N	N	N	
		3	MUD	1 × 10^6^/kg	cryo	N	N	IR	IR	-	N	N	N	
		4	MUD	1 × 10^6^/kg	cryo	N	N	IR	IR	-	N	N	N	
		5	MUD	1 × 10^6^/kg	cryo	N	N	IR	NR	-	N	N	N	

*ALE* alemtuzumab, *ATG* equine anti-thymocyte globulin, *Cryo* cryopreserved cells, *CR* complete response, *CRS* cytokine release syndrome, *DLI* donor lymphocyte infusion, *EBV* Epstein-Barr Virus, *haplo* HLA-haploidentical donor, *HPV* human papillomavirus, *GVHD* Graft-versus-host disease, *IEI* inborn error of immunity, *IR* incomplete response, *MUD* 10/10 HLA-matched-unrelated donor, *MSD* HLA-matched sibling donor, *mMUD* 7/8 HLA-matched-unrelated donor, *N* no, *N/A* not applicable as these patients had full myeloid chimerism at time of DLI, *NE* not evaluable due to too few white blood cells numbers to run chimerism assay, *NR* no response, *PR* progression of disease.

^a^This patient is listed twice as DLI was given for both mixed chimerism and infection (virally-driven squamous cell carcinoma of hands, feet, thighs) who had stability of both viral lesions and chimerism after each DLI.

The majority of the DLIs were unconditioned (*n* = 28), and of the 10 that received conditioning, 5 received corticosteroids alone, 4 received chemotherapy and corticosteroids, and 1 received chemotherapy alone (Table 2 and Table 3). In four patients <120 days post-transplant at the time of DLI, ongoing GVHD prophylaxis with sirolimus (*n* = 5 DLIs) or tacrolimus (*n* = 1 DLI) was continued after DLI (Table 3). Most DLI products were cryopreserved (*n* = 29), while 9 were given from a fresh collection. DLI doses ranged from 1 × 10^5^ to 1 × 10^7^ T cells/kg.

**Table 3:** Characteristics of each DLI.

		Donor type				Dose of DLI						Cryopreserved or fresh		G-CSF-primed*		Immunosuppression after DLI		Conditioning		
Toxicity of DLI, *n* (%)	Total DLIs (*n* = 38)	Haplo (n = 21)	MUD (*n* = 14)	mMUD (*n* = 1)	MSD (*n* = 2)	1 × 10^5^ (*n* = 3)	5–6 × 10^5^ (*n* = 12)	1 × 10^6^ (*n* = 10)	2 × 10^6^ (*n* = 4)	5 × 10^6^ (*n* = 3)	1 × 10^7^ (*n* = 6)	Cryo (*n* = 29)	Fresh (n = 9)	Yes (*n* = 13)	No (*n* = 25)	Yes (*n* = 6)	No (*n* = 32)	Chemotherapy^c^ (*n* = 5)	Steroids only (*n* = 5)	None (*n* = 28)
Acute GVHD, any grade	3	2	1	0	0	0	1	0	0	1	1	3	0	2	1	0	3	0	2	1
Chronic GVHD	1	1	0	0	0	0	1	0	0	0	0	1	0	1	0	1	0	0	0	1
Cytokine release syndrome	3	3	0	0	0	0	2	0	0	1	0	3	0	3	0	0	3	0	0	3
Response To DLI^a^																				
Attainment of full donor myeloid chimerism post-DLI (*n* = 19)	1	1	0	0	0	0	1	0	0	0	0	0	1	0	1	0	1	0	0	1
Attainment of full donor T-cell chimerism post-DLI (*n* = 24)	3	3	0	0	0	0	2	0	0	1	0	3	0	3	0	0	3	0	1	2
Stability of chimerism (*n* = 27)^b^	15	5	10	0	0	2	6	6	1	0	0	11	4	0	15	1	14	0	1	14
Complete remission of malignancy (*n* = 9)	1	0	0	1	0	0	0	1	0	0	0	1	0	1	0	0	1	0	0	1
Mixed response/partial malignancy control (*n* = 9)	3	2	0	0	1	0	0	1	1	0	1	2	1	2	1	0	3	1	0	2
Control of infection (*n* = 7)	7	2	5	0	0	1	2	4	0	0	0	6	1	1	6	1	6	0	0	7
No significant clinical effect (NR or PD)	13	8	4	0	1	0	2	2	2	2	5	11	2	6	7	4	9	3	3	7

*DLI* donor lymphocyte infusion, *Haplo* HLA-haploidentical donor, *MUD* 10/10 HLA-matched-unrelated donor, *mMUD* 7/8 HLA-matched-unrelated donor, *MSD* HLA-matched sibling donor, *Cryo* cryopreserved, *NR* no response, *PD* progression of disease.

*excess cells from a PBSC graft were aliquoted and cryopreserved for future DLI use.

^a^1 patient received 5 MUD DLIs for infection and mixed chimerism and thus these DLIs are counted in each category.

^b^1 patient had stable myeloid chimerism but had too few cells to evaluate CD3 chimerism after his last DLI.

^C^ Patients who received steroids with chemotherapy were included in this group.

### Toxicity

**Fig. 1:**
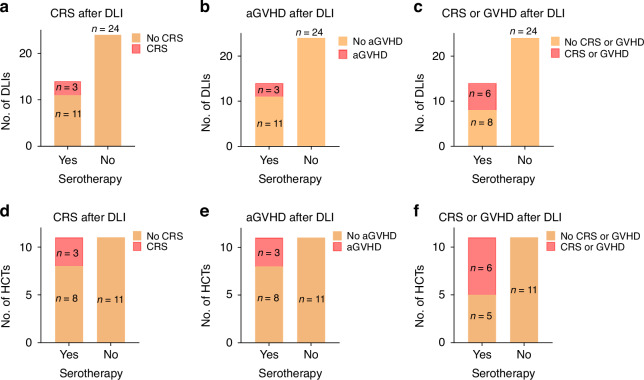
Toxicity of DLI. **a**–**c** Toxicity of DLI based on occurrence of **a** cytokine release syndrome (CRS), **b** acute GVHD (aGVHD), or **c** either CRS or acute or chronic GVHD after each individual DLI. **d**–**f** Toxicity is also illustrated per hematopoietic cell transplant (HCT) based on occurrence of **d** CRS, **e** acute GVHD or **f** any CRS or acute or chronic GVHD, showing that CRS or GVHD occurred only in those that received serotherapy prior to HCT.

Out of 38 DLIs administered, we observed only 3 cases of acute GVHD (one grade II and two grade III), 3 cases of cytokine release syndrome (CRS), and one case of chronic GVHD in a total of 6 patients (Fig. 1, Table 2). All six of the patients with toxicity received serotherapy as part of their conditioning (2 received alemtuzumab and 4 received equine ATG) (Fig. 1, Table 2).

Two different patients had other immune phenomena occur later post-DLI: juvenile dermatomyositis with systemic lupus erythematosus 1 year after the most recent DLI and vitiligo 149 days following the first DLI.

### Efficacy

**Fig. 2:**
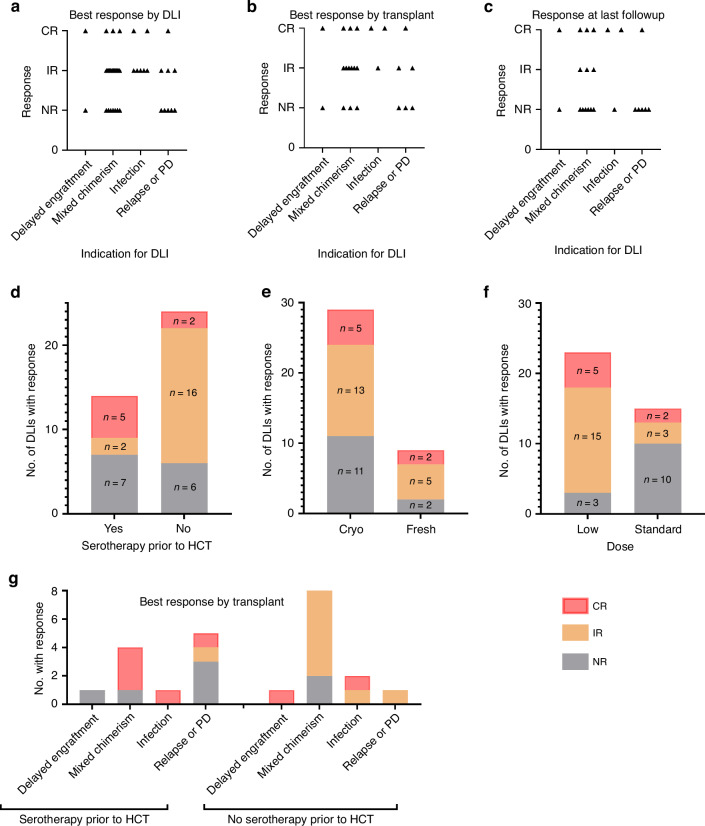
Response to DLI. **a**–**c** Response to DLI with respect to indication for DLI is shown as (**a**, **b**) best response by **a** DLI or **b** transplant, and **c** response at last follow-up for each patient. **d** Complete responses were most often seen in DLIs given after HCT conditioned with serotherapy, but response was not seemingly affected based on **e** whether the DLI had been cryopreserved (cryo) vs. fresh or **f** whether a low-dose or standard-dose DLI was given. Standard doses of DLI were considered to be ≥1 × 10^6^ for HLA-partially-mismatched donors or 1 × 10^7^ for HLA-matched donors. **g** Best response is shown per patient based on indication for DLI and whether serotherapy had been used as conditioning for the HCT after which DLI was given. CR complete response, IR incomplete response, NR no response, PD progressive disease.

**Fig. 3:**
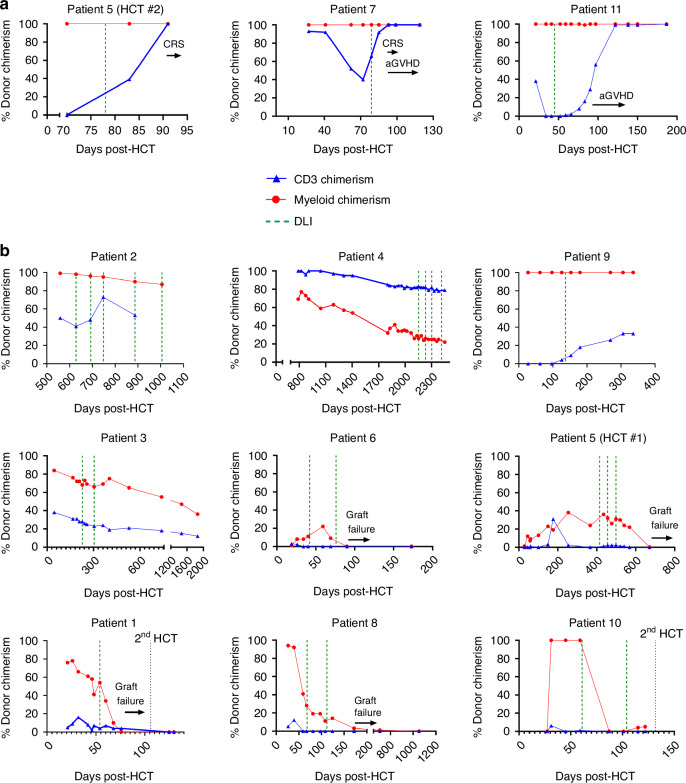
Responses to DLI of Patients with Mixed Chimerism Only in Those Who Had Received Pre-Transplant Serotherapy. DLI was given for mixed chimerism after 12 HCTs, of which **a** only three resulted in complete donor chimerism. All three of these patients had received pre-HCT serotherapy. **b** Of 9 patients who did not achieve complete donor chimerism after DLI, only one (patient 1) received serotherapy prior to transplant. 4 patients achieved stable chimerism (patients 2, 3, 4, and 9; the response of patient 3 was stable only after the first DLI with progressive chimerism decline after the second DLI) and 5 patients had secondary graft failure (patients 1, 5 with 1st HCT, 6, 8, and 10), although patient 6 did have stability of chimerism after his first DLI and patient 5 did have stability of chimerism after her first two DLIs before both patients exhibited secondary graft failure following their final DLIs. Patient numbers shown correspond to the numbering used in Table 2.

The success of each DLI was assessed based on the indication. For six out of the 12 HCTs (50%) in which DLI was given for mixed chimerism, there was no sustained response [35], with 5 of these patients experiencing secondary graft failure despite DLI (Figs. 2 and 3, Table 2); three patients had incomplete responses with two patients continuing to have stable mixed chimerism for >1 year and another having uptrending but low (33%) donor T-cell chimerism 6 months later at the time of death from relapsed leukemia. Of the 6 patients treated for relapsed/progressive hematologic malignancy, one patient had complete response, 2 patients had partial response (duration of response 57 and 102 days), and 3 had no response. Among the two patients given DLI for delayed primary engraftment (mixed chimerism and absolute neutrophil count <500 cells/µl), one promptly engrafted 5 days after DLI administration (day +40) [36], while the other had loss of transient mixed chimerism and confirmed primary graft failure.

**Fig. 4:**
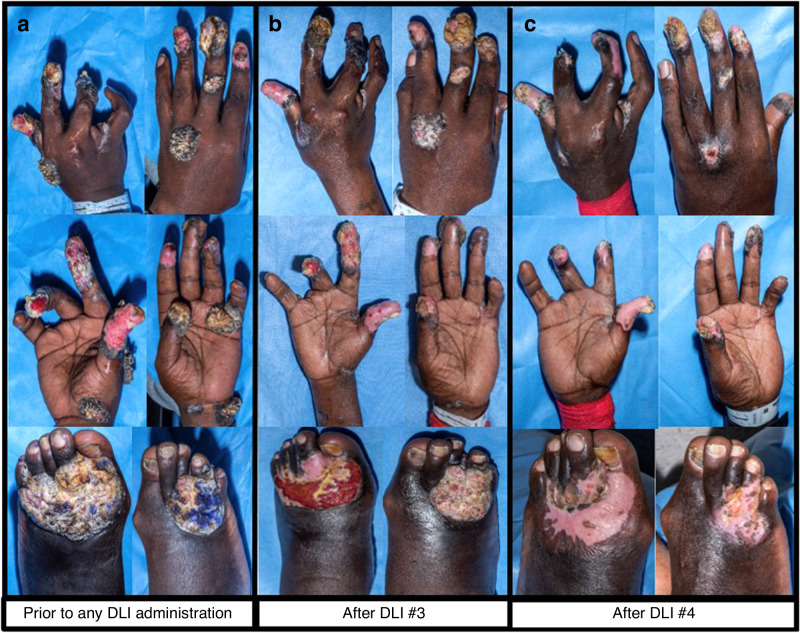
Transient regressions of HPV-associated squamous cell carcinoma (SCC) of the hands and feet in patient 2. A patient with idiopathic CD4 lymphopenia had HPV-associated SCC lesions affecting the hands and feet. **a**–**c** Evolution of these lesions are shown in relation to DLI administration. Overall, there were transient regressions seen in these lesions as shown after **b** DLI #3 and **c** DLI #4 with adjunctive surgical debridement as compared to (**a**) SCC lesions shown before any DLI administration, along with subjective improvements noted by the patient such as slower growth and decreased pain.

By contrast, all patients who received DLI for infection resultant from poor immune reconstitution had response: 1 incomplete and 2 complete. Our previously mentioned patient (Patient #2 in Table 2) with incomplete response was a subject with idiopathic CD4 lymphopenia [37] who had HPV-associated SCC existing prior to HCT and post-HCT had both mixed chimerism and poor immune reconstitution. He received a total of 5 DLIs, and, with each DLI and concurrent surgical debridement, we noticed partial regression of the SCC on his hands and feet (Fig. 4) in the absence of any notable toxicity. However, before a more definitive solution (second HCT or systemic therapy for SCC) could be attained, he was incarcerated and his SCC progressed with time. A second patient (Patient #20 in Table 2), who was CMV-naïve pre-HCT, developed CMV pneumonitis requiring prolonged extra-corporeal membrane oxygenation and need for continuous renal replacement therapy 7 months post-HCT after primary CMV infection. Given her dire clinical situation, on day +236, she received DLI from her CMV-seronegative donor to help control her CMV infection, and she recovered completely from the infection. A third patient (Patient #21 in Table 2) had high-level adenoviremia at 1 month post-HCT which was fully controlled after DLI administration and concurrent de-escalation of GVHD prophylaxis.

In evaluating cofactors that may correlate with response to DLI, pre-transplant serotherapy seemed to correlate both with response and associated toxicity (Figs. 1 and 2), with a larger number of complete responses noted after DLI after transplants with conditioning serotherapy (35% vs 8.3% of DLIs led to complete response) and all CRS and GVHD events occurring after DLI in patients receiving pre-transplant serotherapy as above (43% vs. 0% of DLIs led to GVHD or CRS).

## Discussion

We have found that patients receiving PTCy-based HCT and therapeutic DLI had overall low rates of GVHD or CRS with only three cases of acute GVHD (14% of patients, 8% of DLIs), three cases of CRS (14% of patients, 8% of DLIs), and one case of late-onset chronic GVHD among six patients. All patients who had acute GVHD, CRS, or chronic GVHD had received serotherapy prior to HCT as part of their conditioning regimen. The rates of complete response to DLI by indication were low at 11% for relapse and 15% for mixed chimerism or delayed engraftment, but we did see some apparent efficacy, albeit with small numbers, when using DLI for infection with two of three patients having complete response (incomplete response only in treatment of HPV-associated SCC). Of patients receiving serotherapy-containing pre-HCT conditioning, there appeared to be more complete responses overall, which was particularly apparent for the treatment of mixed chimerism, than when compared with patients not receiving serotherapy.

Although our numbers of patients are limited, the rates of acute GVHD from DLI appear low compared with other studies employing therapeutic HLA-matched or HLA-partially-mismatched DLIs without PTCy (35–60% acute GVHD) [3, 4, 7, 8, 9, 12]. This result is consistent with our preclinical data showing that PTCy induces Treg-mediated suppressive mechanisms that minimize GVHD induction by subsequent cell infusions [15, 16, 20]. The rates of GVHD after DLI in our patients are even lower than those reported in some other studies of PTCy-treated patients [22–24]. It is possible that this is attributable to the lack of pre-DLI conditioning in many of our patients, as lymphodepleting chemotherapy prior to DLI is associated with higher rates of GVHD [11, 12, 14, 38, 39]; even so, in our limited dataset, we did not see any seeming correlation between pre-DLI conditioning and toxicity or efficacy. Other factors could contribute such as the use of sirolimus (conducive to T_reg_ recovery) rather than tacrolimus for 73% of our patients or the use of lower-dose DLI in many cases. Many DLIs were administered for mixed chimerism or infection wherein a clinical benefit was intended but there was a strong interest in minimizing risk for GVHD; therefore, a low starting dose was often used for the first DLI with subsequent doses being escalated. Even so, the dosing of DLI did not seem to correlate strongly with toxicity or response (Table 2 and Fig. 2f). Lastly, many cryopreserved and/or granulocyte-colony stimulating factor-primed (aliquoted from original cryopreserved PBSC graft) DLIs were used; although it is possible that this may have contributed to our overall low rates of response and toxicity [40], all toxicity events actually occurred in patients receiving cryopreserved DLI so this possibility is unlikely.

Consistent with the attenuation of alloreactivity, the therapeutic efficacy of these DLIs also was limited, except for control of infection (a non-alloreactive response). DLI is heterogeneously effective across diseases, with very high response rates in CML, but much lower response rates in AML and very poor responses in ALL or B-cell lymphomas. Most of our patients treated for relapse with DLI had T-cell diseases (adult T-cell leukemia/lymphoma, anaplastic large cell lymphoma, hepatosplenic T-cell lymphoma, NK/T-cell lymphoma); DLI has a relatively high response rate of 44–66% in adult T-cell leukemia/lymphoma and peripheral T-cell lymphoma [41, 42], but uncertainty of response for many other T-cell diseases. The seemingly low rate of response in our cohort may be due to the heterogeneity of diseases treated with DLI and the variable susceptibility of these diseases to immune-mediated therapy. Additionally, we were unable to evaluate for loss of HLA-haplotype as a potential mechanism of immune evasion in all patients who received an HLA-haploidentical DLI (one non-responding patient was confirmed not to have had HLA loss), and this could have contributed to the low response rate. Lastly, the lack of lymphodepleting conditioning immediately prior to DLI for most of our DLIs again may have contributed to the low response rate seen [9, 11–14].

Yet, the attenuated response to DLI seen appears partially modifiable by pre-HCT serotherapy as all six patients with GVHD or CRS had had serotherapy (ATG or alemtuzumab) as part of pre-HCT conditioning. A prospective trial investigating prophylactic DLI given to patients with AML or ALL receiving alemtuzumab as pre-transplant conditioning also noted a high rate of GVHD (7 out of 16 patients) with 3 patients dying from steroid-refractory grade III acute GVHD despite low-dose DLI in two of these patients (<1 × 10^6^ T cells/kg) [43]. These results lead us to postulate that pre-transplant serotherapy may potentially disrupt the suppressive mechanisms, particularly expansion of T_regs_, induced by PTCy, interfering with their protection against GVHD inducible by subsequent T-cell infusions [15, 20]. In fact, when T_regs_ are higher post-DLI as in the case of NK-cell-enriched infusions, there was markedly less GVHD than the same platforms without DLI in which levels of T_regs_ were lower [44].

The apparent therapeutic response to DLI when used for infectious indications was encouraging. However, it is impossible to definitively attribute enhanced immune reconstitution/therapeutic effect to the DLI only and not just to resolution with time or control of the infection by donor immune cells previously received during the transplant. Moreover, it has been previously demonstrated that DLI has effects on donor immune cells already present in the recipient [45], so the distinction becomes even more challenging. Lastly, some patients were receiving concurrent anti-viral therapy or had decreases in immunosuppression, which may have contributed.

There are several limitations to our retrospective study. Although our study has comparable patient numbers as other reports of DLI for PTCy-treated patients [22–24], our study is still modest in size. Moreover, there is a heterogeneous patient population with diverse indications for DLI and the primary HCT platforms varied including different donor types. Additionally, DLI sources were not uniform as some were aliquoted from mobilized PBSC grafts while others were harvested from separate peripheral blood collections without filgrastim exposure. DLI doses ranged from 10^5^ to 10^7^ T cells/kg and the time to DLI from HCT varied greatly as well from day +31 to day +2411. This variability in disease type; HCT platform; DLI dose, source, and timing; and HLA-matching limits our ability to draw more definitive generalized conclusions from our data. Moreover, our institution is a research hospital, with all patients transplanted on a research study, each of which utilize proscribed transplant platforms for particular patient populations. Serotherapy was a component of the transplant platform for only two of the five studies included in this study, and thus there is the possibility of selection bias particularly related to disease types and treatment history among patients that received serotherapy (these two studies were for patients with T-cell lymphoma [NCT03922724] or immune dysregulation/inborn errors of immunity (IEI) [NCT03663933], whereas two of the three studies not using serotherapy were for all hematologic malignancies (NCT03983850 and NCT04959175, both of which largely enrolled acute leukemias) and the third (NCT02579967) was for IEI). Nevertheless, the transplant platform is nearly identical between the two serotherapy studies and the serotherapy-free IEI study; only three of the 22 HCTs occurred on the other two protocols that enrolled all types of hematologic malignancies. While we considered applying formal statistical analysis to assess the effects of serotherapy or other factors on outcomes, the substantial interpatient and DLI characteristic heterogeneity made such formal statistical comparisons in this small sample size not meaningful, and thus we opted to provide only descriptive statistics.

Despite the wide variety of therapeutic indications, diseases, and DLI conditions of our cohort, it does appear that DLI administered after PTCy has low rates of GVHD, but strategies to improve DLI efficacy are needed. Given encouraging results with prophylactic DLIs, it is possible that administering DLIs earlier might promote activity before the suppressive mechanisms are fully established [46, 47]. Our preclinical data, however, suggest that this window may be brief in PTCy-treated recipients [48]. Since our DLIs for relapse were all administered beyond post-HCT day +31, we may have missed a window of efficacy for the DLI. We are testing this potential for very early prophylactic DLI to effect a better anti-tumor response for high-risk malignancies in an ongoing clinical trial (NCT05327023). Alternative approaches include NK-cell-based therapies, which have the advantage of minimal risk of GVHD [49, 50]. Nevertheless, we have demonstrated that T-cell-containing DLI with PTCy also are safe with low rates of alloreactivity, and thus we may not need to limit our therapeutic options for post-HCT relapse to NK-cell therapies alone.

Even so, the safety of DLIs given to our patients is a double-edged sword. The therapeutic intent of DLI, except for infection, is to induce a better graft-versus-host response against residual malignancy or normal host hematopoiesis. Yet, DLI, while safe, may have low efficacy in treating relapse or stabilizing graft function unless given in the setting of serotherapy-containing pre-HCT conditioning. Thus, novel therapies are needed in PTCy-treated patients to exert a stronger anti-tumor or anti-host hematopoiesis response and overcome the immunosuppressive environment induced by PTCy but still not incite GVHD.

## Data Availability

The datasets generated during and/or analyzed during the current study are available from the corresponding author on reasonable request.
